# Supplementary material for: Perioperative Predictive Factors for Tumor Regression and Survival in Non-Small Cell Lung Cancer Patients Undergoing Neoadjuvant Treatment and Lung Resection
Source: Cancers (Basel). 2024 Aug 20;16(16):2885. doi: 10.3390/cancers16162885 (PMC11352403; doi:10.3390/cancers16162885)
Supplement: Supplementary file 1 [file cancers-16-02885-s001.zip › cancers-3125131-supplementary.pdf]

Supplementary Table S1. Preoperative laboratory parameters in patients undergoing neoadjuvant therapy and surgical resection classified by histology of primary tumor.

Parameters preoperatively (median, quartiles [1st; 3rd])	LUAD n = 58	SQCA n = 37	P-value
Blood counts			
Leukocytes (/nL)	6.15 [4.58; 7.30]	5.60 [4.35; 7.90]	0.7923
Erythrocytes (/pL)	3.74 [3.48; 3.91]	3.56 [3.33; 4.01]	0.7688
Hemoglobin (g/dL)	11.9 [11.10; 12.43]	11.6 [10.85; 12.60]	0.8396
Thrombocytes (/nL)	240.5 [204.0; 288.25]	221.0 [198.5; 268.5]	0.3046
Clinical chemistry			
CRP (mg/dL)	0.20 [0.10; 0.60]	0.50 [0.10; 1.30]	0.0206
Creatinine (mg/dL)	0.90 [0.78; 1.00]	1.0 [0.80; 1.10]	0.1304
Albumin (g/dL)	4.27 [3.90; 4.46]	4.00 [3.60; 4.35]	0.0281
LDH (IU/L)	200.0 [169.8; 244.0]	188.0 [174.0; 211.0]	0.2323

For continuous variables, non-parametric Mann-Whitney U test was performed. P-values < 0.05 are statistically significant. Abbreviations: LUAD: lung adenocarcinoma; SQCA: squamous cell lung cancer; CRP: C-reactive protein; LDH: lactate dehydrogenase.

Supplementary Table S2. Used drugs regimens in patients undergoing neoadjuvant therapy and surgical resection classified by histology of primary tumor

Used drugs regimens for neoadjuvant therapy	LUAD n = 58 (n,%)	SQCA n = 37 (n,%)	P-value
Platine derivatives + vinca alkaloids	2/58 (3.4%)	1/36 (2.8%)	1.000
Platine derivatives + taxane + vinca alkaloids	26/58 (44.8%)	28/36 (77.8%)	0.0017
Platine derivatives + taxane	9/58 (15.5%)	4/36 (11.1%)	0.5475
Platine derivatives + antimetabolites	7/58 (12.1%)	1/36 (2.8%)	0.1480
Platine derivatives + antimetabolites + immunotherapy	6/58 (10.3%)	0/36 (0.0%)	0.0790
Platine derivatives + antimetabolites + vinca alkaloids	3/58 (5.2%)	0/36 (0.0%)	0.2835
Platine derivatives + taxane + immunotherapy	1/58 (1.7%)	2/36 (5.6%)	0.5561
Platine derivatives + taxane + vinca alkaloids + immunotherapy	2/58 (3.4%)	0/36 (0.0%)	0.5223

Platine derivatives + antimetabolites + vinca alkaloids + immunotherapy	1/58 (1.7%)	0/36 (0.0%)	1.0000
Platine derivatives + topoisomerase II inhibitors	1/58 (1.7%)	0/36 (0.0%)	1.0000

For binary variables Pearson chi-square test or Fisher's exact test was performed. P-values < *0.05* are statistically significant. Abbreviations: LUAD: lung adenocarcinoma; SQCA: squamous cell lung cancer

Supplementary Table S3. Binary logistic regression model predicting complete pathologic response in primary lung cancer patients undergoing neoadjuvant therapy and major surgical resections.

**Covariates for tumor regression**	**Exp(B) [95% CI]**	**P-value**
Intraoperative histology (LUAD vs. SQCA)	6.81 [2.34-19.77]	0.0004
Lymph node size in pre-neoadjuvant PET > 1.7 cm	3.86 [1.35-11.10]	0.0119
Relative delta value > 30%	4.54 [1.49-13.84]	0.0079

Abbreviations: LUAD: lung adenocarcinoma; SQCA: squamous cell lung cancer; PET: positron emission computed tomography; Exp(B) = Odds ratio, 95% Confidence interval [lower bound-upper bound]. Relative delta values = ((tumor size before neoadjuvant treatment - tumor size after neoadjuvant treatment) / tumor size before neoadjuvant treatment) x 100.
